# Blood inorganic mercury is directly associated with glucose levels in the human population and may be linked to processed food intake

**DOI:** 10.15761/imm.1000134

**Published:** 2015

**Authors:** Renee Dufault, Zara Berg, Raquel Crider, Roseanne Schnoll, Larry Wetsit, Wayne Two Bulls, Steven G. Gilbert, H.M. “Skip” Kingston, Mesay Mulugeta Wolle, G.M. Mizanur Rahman, Dan R. Laks

**Affiliations:** 1 Food Ingredient and Health Research Institute, Naalehu, Hawaii, USA; 2 Fort Peck Community College, Poplar, Montana, USA; 3 Shepherd University, Shepherdstown, West Virginia, USA; 4 Department of Health and Nutrition Sciences, Brooklyn College of City University of New York, Brooklyn, New York, USA; 5 Institute of Neurotoxicology and Neurological Disorders, Seattle, Washington, USA; 6 Department of Chemistry and Biochemistry, Duquesne University, Pittsburgh, Pennsylvania, USA; 7 Department of Biological Chemistry, University of California Los Angeles (UCLA), Los Angeles, California, USA

**Keywords:** macroepigenetics, fructose, corn syrup, diet, diabetes, online, glucose, NHANES, mercury

## Abstract

**Background:**

The goals of the study were (1) to determine the impact of inorganic mercury exposure on glucose homeostasis; and (2) to evaluate the effectiveness of two community-based interventions in promoting dietary changes among American Indian college students to reduce risk factors for Type-2 Diabetes including fasting glucose, insulin, and mercury levels, weight, and body mass index.

**Methods:**

To accomplish goal one, the National Health and Nutrition Examination Survey (NHANES) dataset was analyzed using a previously published method to determine if there is a relationship between inorganic blood mercury and fasting glucose. To accomplish goal two, ten college students were recruited and randomly assigned to a group receiving the online macroepigenetics nutrition course and the support group for eliminating corn sweeteners. Participants in both groups were assessed for diet patterns, weight, body mass index (BMI), fasting glucose, insulin, and mercury levels. The interventions were implemented over a 10-week period.

**Results:**

Analysis of the NHANES data (n=16,232) determined a direct relationship between inorganic mercury in blood and fasting glucose levels (p<0.001). The participants who took the online macroepigenetics nutrition intervention course significantly improved their diets (p<0.01), and fasting blood glucose levels (p<0.01) while having lower levels of inorganic mercury in their blood compared to the subjects in the group who eliminated corn sweeteners from their diet and participated in the support group. The trend in lower blood inorganic mercury was strong with p=0.052. The participants in the support group who eliminated corn sweeteners from their diet achieved significant weight loss (p<0.01) and reduced their body mass index (p<0.01).

**Conclusion:**

Total blood mercury levels may be influenced by dietary intake of highly processed foods and lower inorganic mercury levels are associated with lower fasting glucose levels. Alternative community-based interventions emphasizing the role food ingredients and toxic substances play in gene modulation and the development of diseases can result in significant dietary improvements and reductions in risk factors associated with type-2 diabetes. A healthier diet can be promoted among community members using a novel online nutrition course. Consumption of corn sweeteners may be a risk factor in the development of obesity.

## Background

American Indians and Alaska Natives are persons having origins in any of the indigenous populations of North America who maintain tribal affiliation or membership [1]. Indian Country is comprised of American Indian (AI) and Alaska Native (AN) communities across the United States of America (USA). Compared with other Americans, AI and AN populations experience a disproportionate burden of chronic disease including liver disease and cirrhosis, diabetes, and heart disease [2,3]. Heart disease and type-2 diabetes (T2D) are the top two leading causes of death in AI and AN communities [2]. Diabetes prevalence rates are increasing among the AI and AN population aged 20 years or older and vary by region from 6.0% among AN adults to 24.1% among AI adults in southern Arizona [4,5]. Obesity is a significant risk factor in the development of both heart disease and T2D and contributes to the high prevalence rates of these diseases in Indian Country [6]. Although excess caloric consumption and a sedentary lifestyle are well known risk factors for obesity, T2D and heart disease, there is increasing evidence to suggest that exposure to toxic environmental substances and depletion of dietary micronutrients may play a significant role in the etiology of these diseases [7–10]. Toxic metal and pesticide exposures and excess consumption of food ingredients known to affect micronutrient status have been linked to the development of diabetes [8–11] through gene-environment interactions [8,10,11].

Adequate dietary intake of micronutrients is necessary to sustain metabolism and tissue function [12]. A recent study reported that concentrations of chromium (Cr), copper (Cu), manganese (Mn), nickel (Ni), lead (Pb) and zinc (Zn) in foodstuffs significantly correlated with that in human blood following intake [13]. In the only published report thus far about the dietary intake of a representative sample of the AI population, many of the AI men and women were not meeting the dietary recommendations for the key micronutrients magnesium (Mg) and Zn [14]. This finding supports the conclusion made a few years earlier by USA Department of Agriculture scientists who reported nearly one half of all Americans one year old and over had inadequate intakes of dietary Mg [15]. Such micronutrient insufficiencies over time may lead to increased risk for heart disease, diabetes, and neurodevelopmental disorders [16–19]. Zn, Mg, and phosphorus (P) losses can occur as a result of excess consumption of high fructose corn syrup (HFCS) and create imbalances in dietary micronutrient status [20,21]. Such imbalances are problematic from a macroscopic gene-environment point of view since dietary micronutrient status can either exacerbate or mitigate the effects of exposure to toxic environmental substances by altering gene function especially in the case of child neurodevelopment and diabetes [17,22,23]. Fructose consumption in dietary conditions of magnesium deficiency induces insulin resistance while lowering PON1 gene activity [22,23]. PON1 gene expression is needed by the body to metabolize organophosphate pesticides known to adversely impact child neurodevelopment [17]. Child and reproductive health and diabetes are all health issues of grave concern to the indigenous populations of North America [24].

Health education can play a role in addressing these health issues if it is delivered in a culturally competent manner. Community based and culturally competent education efforts have made a positive difference in diabetes prevention efforts in minority communities by empowering community members to make lifestyle changes through the acquisition and dissemination of diabetes knowledge [25]. During the previous year, Fort Peck Community College (FPCC) collaborators developed and evaluated the efficacy of a culturally competent online nutrition intervention course [26]. The course was found to be effective in producing healthful dietary changes among community members who completed it [26].

The curriculum of the course is based on the underlying assumption that the prevalence of T2D among the AI population is due to the industrialization of the food supply, dietary exposure to toxic substances, and a general lack of knowledge among community members as to the epigenetic role food ingredients, nutrition, and invasive toxic substances play in the development of diseases. Epigenetic changes impacting human metabolism and health can occur through nutrition via dietary intake of methyl donating nutrients such as choline and betaine [27–29]. Macroepigenetics is a theoretical, consumer friendly approach that allows laypeople to consider how factors of nutrition, environment and gene expression interact to contribute to the development or prevention and inheritance of disease [17]. In accessing the macroepigenetics nutrition intervention course, participants are provided numerous opportunities to review research on the role nutritional factors and invasive toxic substances in the food supply play in gene modulation making them more susceptible to diabetes and other disease conditions. Participants learn that genes turn on and off in response to diet to produce the hormones and proteins needed by the body to regulate metabolism. They also learn that exposure to toxic substances such as mercury and fructose interfere with body metabolism. Participants finishing the online nutrition intervention course earn 3 units of science credit at FPCC. In this study, we wanted to determine if dietary changes resulting from participating in the online nutrition intervention course could result in reductions in risk factors associated with T2D including fasting blood glucose, insulin and mercury levels, weight, and body-mass-index (BMI).

Recognizing a one size fits all approach rarely works to resolve any health issue; we also wanted to offer an alternative intervention for individuals wishing to improve their health status by eliminating their consumption of corn sweeteners. Synthesized from corn starch [30] the targeted corn sweeteners included high fructose corn syrup (HFCS), corn syrup, modified corn starch, dextrose, maltodextrose, maltodextrin, and fructose. From a macroepigenetic perspective, HFCS is an invasive toxic substance for at least three different reasons. Its consumption by humans can result in dietary mercury exposure [31, 32], and insulin resistance [33–35] and reduced PON1 gene activity in rats [22]. PON1 is the gene responsible for producing the paraoxonase enzyme required for breaking down the toxic organophosphate pesticide residues [22] found in wheat, corn, and wheat products [36]. Patients with T2D who have insulin resistance also have lower paraoxonase activity [37]. While it is unclear how decreased PON1 activity contributes to the development of T2D, there is evidence to suggest that inorganic mercury exposure plays a role in insulin resistance. Inorganic mercury exposure in the mercuric chloride (HgCl_2_) form is suspected to be a contributing factor to the onset of insulin resistance by interfering with genes that regulate glucose homeostasis [38,39]. Exposure to inorganic mercury in the environment has also been found to contribute to the development of insulin resistance in non-diabetic humans by interacting with other toxins, such as dioxin [28,40].

Mercury exposure can be determined through the analysis of a variety of tissues to include blood, urine, finger or toe nails, breast milk and hair [41]. Many studies have measured total Hg in blood without distinguishing the forms of mercury found in the blood [41]. This study was focused on determining the form of mercury exposure from consumption of processed foods. Inorganic mercury may enter food products during the various manufacturing processes. For example, mercury cell chlor-alkali chemical products are used extensively in food processing and always contain inorganic mercury residues. Vegetable oil products manufactured using the common *alkali* refining process may present a moderate risk of mercury contamination [42]. The mercury cell chlorine used to bleach flour is expected to contain a small amount of mercury residue [43]. The corn starch used to manufacture the corn sweeteners in the HFCS product line is treated purposely with inorganic HgCl_2_ as part of the manufacturing process to inhibit endogenous starch-degrading enzymes [30]. It is thus reasonable to suggest that consumers are routinely exposed to non-elemental inorganic mercury (I-Hg) when they consume heavily processed foods, including corn sweeteners. Our justification for adding the support group intervention to help students and community members eliminate corn sweeteners from their diet is based on the concept that consumption of corn sweeteners is both a known and potential source of inorganic mercury exposure [31,32] and a potential factor in the development of insulin resistance [44].

## Methods

The first goal of this study was to determine the impact of inorganic mercury exposure on glucose homeostasis through an analysis of the NHANES dataset.

### NHANES dataset analysis

The NHANES is a dataset of measured outcome variables such as biomarkers for the target population of the non-institutionalized, civilian United States population. The NHANES survey is generated in data groups of separate 2-year clusters. Our study analyzed the NHANES datasets combining survey clusters from 1999–2012 and was focused on the following biomarkers: fasting glucose, blood inorganic mercury (I-Hg), organic mercury, and insulin. In addition to analyzing blood I-Hg as a continuous variable, we also assessed its associations as a binary variable.

The rationale and methods to generate the binary variable blood I-Hg detection (I-Hg detect) are detailed in Laks [45]. We set a constant value across survey years for the limit of detection (LOD) based on the first survey years limit of detection (0.4 ug/L) and used that as a standardized baseline. Thereby, we generated a binary variable for I-Hg detection, where 0=no detection (below 0.4 ug/L) and 1=positive detection (above or equal to 0.4 ug/L). This maintained a standardized limit of detection for all survey groups. This coding procedure was followed because most values for blood I-Hg fell below the LOD and were assigned estimate values that changed across survey years. Thus, LBXIHG, the NHANES variable for I-Hg is not an optimal continuous variable but may be better assessed as a binary variable of detection/no detection. To compensate for the changing estimate values below the LOD, values below our assigned and uniform LOD of 0.4 ug/L were assigned a constant estimate value of 0.3 ug/L which was the estimate value used for the initial 1999–2000 survey set. We stress that this was done only to estimate the rate of I-Hg detection in blood. In order to determine associations with blood I-Hg concentration we also used the primary NHANES continuous variable, LBXIHG (blood I-Hg). We found comparable results for associations with both I-Hg detection and I-Hg mean concentration. In addition, we generated a value for blood organic mercury, CH_3_Hg by subtracting blood I-Hg (LBXIHG) from total blood mercury (LBXTHG).

This study analyzed both the raw population and the survey weighted population in order to detect robust associations. The survey-weighted population extends the inferences based on our findings to the U.S. population and utilizes a weighting adjustment as outlined in the NHANES website. The stratum and PSU variables assist in estimating variances in order to reflect the design structure of the NHANES survey. In STATA the 1999–2012 NHANES combined data set is weighted for survey analysis by: svyset(pw=WT99–2012), psu(sdmvpsu), strata(sdmvstra) where WT99–2012 is the combined weight calculated above. We chose to report as significant only those associations with p-values less than 0.01 in the survey-weighted population.

The NHANES population analyzed was the full NHANES population available with measured blood I-Hg levels. However, we also adjusted for potential confounders and used multivariate analysis to adjust for age, race and gender. We used linear regression and logistic regression models when appropriate. Statistical analysis of the NHANES dataset was performed using STATA 8.0 (StatCorp).

### Health education interventions

The second goal of this study was to evaluate the effectiveness of two health education interventions in reducing risk factors for T2D: the online macroepigenetics nutrition course (MAC) and the support group for corn sweetener elimination (CSE). Participants in both groups were assessed for weight, BMI, fasting glucose, insulin, and mercury levels. The interventions were implemented over a 10-week period.

This study was conducted at the Fort Peck Community College (FPCC) in compliance with a protocol approved by a third party non-profit Institutional Review Board (IRB), the BioMed IRB [46]. The tribe did not have their own IRB at the time but the Fort Peck Tribal Executive Board passed a resolution in support of the members participating in a macroepigenetic study of changes in health status following the nutrition education intervention program at FPCC. Located in Popular, Montana on the Fort Peck Indian Reservation, FPCC is a tribally controlled community college chartered by the government of the Fort Peck Assiniboine and Sioux Tribes. The macroepigenetics nutrition intervention course (MAC) and pretest and posttest food frequency survey questionnaire were delivered on line via the non-profit Food Ingredient and Health Research Institute (FIHRI) website [47] to the FPCC students. The corn sweetener elimination (CSE) support group intervention was delivered by FPCC teaching staff. While ten community members initially enrolled in the study only nine successfully participated in one of the alternative interventions offered during the study.

The nursing staff at the Northeast Montana Health Services (NEMHS) Riverside Clinic screened ten participants and randomly assigned half of them to the CSE intervention group. The five participants in the CSE group were asked to eliminate corn sweeteners in the HFCS product line from their diet and participate in a support group. Screening was conducted to ensure that participants, regardless of the intervention assigned, were at least 20 years of age and not on any medication except birth control. The nursing staff was also responsible for collecting weight and height measurements, and blood samples for biomarker analysis.

Community members participating in either the online intervention course (MAC) or the CSE group received a monthly stipend of $200 for each month of successful participation. Success in the nutrition intervention course (MAC) was determined by completed homework submissions, participation in the online peer group discussion forum, and completion of a culminating final project. Success in the support group (CSE) was determined by meeting weekly with the support group coordinator. The stipend was provided to compensate the community members for their time in completing the online course work with the embedded pre and post food frequency survey or meeting weekly with the support group coordinator, and providing blood samples for analysis and height and weight measurements for determining BMI.

### MAC intervention course

The online MAC course consisted of ten modules of culturally competent instruction delivered over ten weeks at the FIHRI website which also provides an outline of the course content [47]. Each of the five participants in the course was provided with their own unique password to access the modules of instruction. The curriculum provided opportunities for independent research, collaboration, and peer group interactions to discuss lessons learned. For example, during the third module of instruction, participants were required to access the United States Department of Agriculture (USDA) Food Availability Data System and determine the average per capita availability and consumption for various foods consumed in the US [48]. After compiling their data, they were then asked to interpret the changes in commodity consumption over time and how these changes might contribute to the development of modern diseases. Table 1 provides an example of the data the participants gathered for sugars and vegetable oils from 1970 to 2010. The participants were able to compare the per capita American consumption rates of high fructose corn syrup, cane and beet sugar, total corn sweeteners, and vegetable oils over time. Using the USDA data, the participants determined that from 1970 to 2010, cane and beet sugar consumption decreased 35% while high fructose corn syrup consumption increased 9,467%. During the same period of time, per capita vegetable oil consumption increased 248%. In their online forum, they discussed the potential impact of this increased high fructose corn syrup and vegetable oil consumption on Indian health. In a later module, the participants reviewed peer reviewed journal articles and learned that fructose consumption impacts PON1 gene activity which may create conditions of oxidative stress leading to the development of insulin resistance and T2D [22].

To evaluate whether or not the curriculum was effective in reducing participant consumption of food commodities and ingredients that lead to the development of chronic diseases, a one-group pretest-posttest survey was designed and administered online prior to and after receiving the instruction over the 10-week long intervention period. The IRB approved survey was constructed and delivered using the online Survey Monkey tool [49]. Food frequency questions were modeled after those used by the National Cancer Institute [50] to query dietary intake during the past month. Using the same format we developed three additional questions to determine the intake of organic flour, organic vegetables and fruit, and organic processed foods (crackers, bread, and cereal).

### CSE support group intervention

Participants in the CSE support group were provided the following:

- A shopping guide with food ingredients to avoid including HFCS, corn syrup, modified corn starch, dextrose, maltodextrose, maltodextrin, and fructose.
- Instructions on the importance of reading food ingredient labels as opposed to “nutrition facts.”
- Field trip to grocery store with one-on-one support instruction on reading food ingredient labels.
- Alternative recipes for preparing favorite meals without corn sweetener ingredients. For example, corn syrup was eliminated as an ingredient in making Indian fry bread.

### Participant blood sample analyses

Pre- and post-intervention blood samples were analyzed successfully to determine fasting glucose and insulin levels using standard methods at the local clinical laboratory. Insulin and glucose measurements for each participant were entered into the Hepatitis C Society’s online homeostasis model of assessment for insulin resistance (HOMA-IR) calculator [51] for determining the degree of insulin resistance.

The pre-intervention blood testing was performed at the Mayo clinic but because measurements were reported as <1, 1 or >1 xg/g mercury (Hg), they were not useful for our purpose which was to measure lower detectable Hg exposures. Post-intervention Hg samples were sent to Duquesne University for analysis using a method developed for and used by the Centers for Disease Control and Prevention (CDC) [52]. These samples were analyzed for total Hg based on microwave enhanced sample digestion (EPA Method 3052) with direct isotope dilution mass spectrometry, D-IDMS (EPA Method 6800) using Agilent 7700 inductively coupled plasma mass spectrometry (ICP-MS). In addition, two of the post intervention blood samples were analyzed for Hg^2+^ and CH_3_Hg^+^ based on microwave enhanced extraction (EPA Method 3200) with speciated isotope dilution mass spectrometry, SIDMS (EPA Method 6800) using Agilent 7890 gas chromatograph (GC) connected to Agilent 7700 ICP-MS. The method was capable of detecting Hg species down to ng/g levels. Each of these EPA numbered methods were developed by the same research laboratory at Duquesne University and validated and published by the Environmental Protection Agency (EPA) under Resource Conservation and Recovery Act (RCRA) statues [53].

### Statistical analysis of pilot study data

Statistical analysis was performed using Excel software. Results are expressed as mean difference and standard deviation (SD). A p value of <0.01 is considered significant. A one tailed t-test analysis was conducted to compare the mean of the difference pre and post within each intervention group. In the comparison of the post intervention mercury levels, the online SISA analytical tool [54] was used to conduct the t-test of the means for the two unequal samples (n=5 and n=4). The diet score data in Table 2 was tallied using a scoring method of providing one point for each item if the participant in the MAC intervention course reported a diet habit consistent with the instruction. For example, in scoring each question on highly processed food consumption, participants reporting less consumption were awarded one point. The higher score in this category indicates less consumption of highly processed foods. Conversely, the higher score in the whole foods (minimal processing) and/or organic products category indicates more consumption of foods less likely to contain invasive toxic substances introduced through processing.

### Results of NHANES dataset analyses

**Figure 1:**
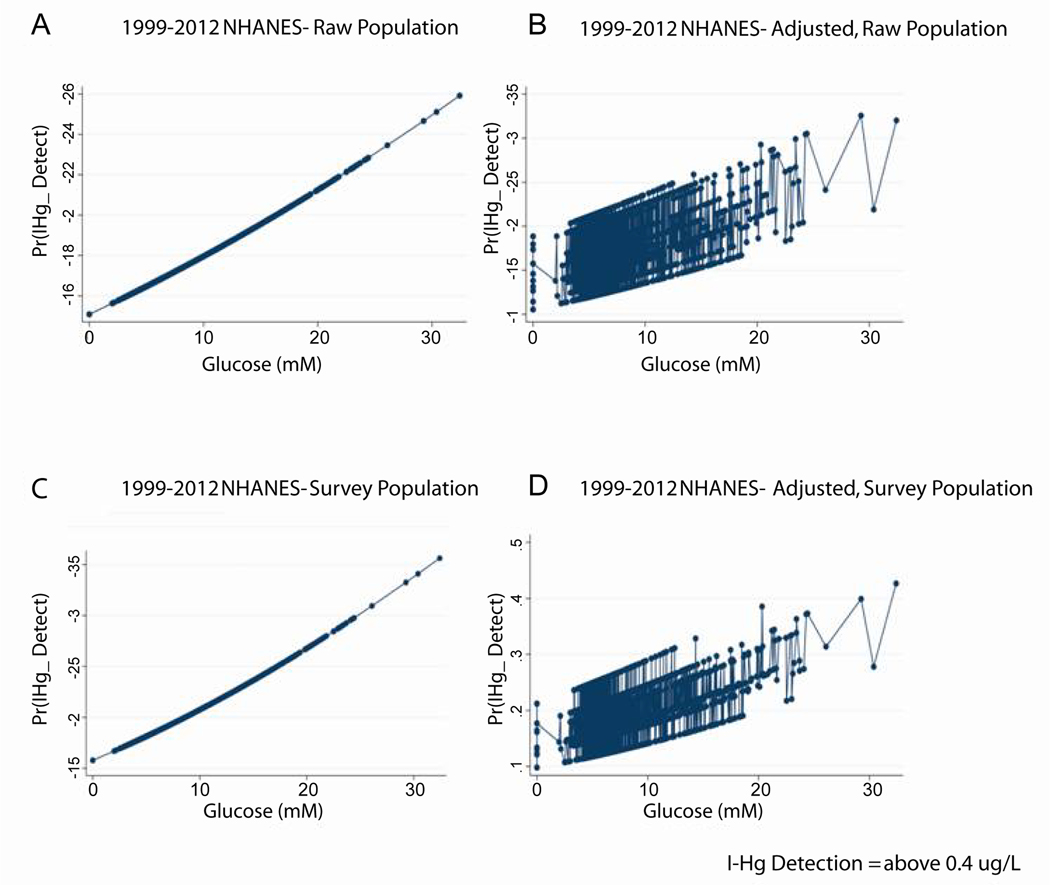
Inorganic Hg (I-Hg) detection is directly associated with fasting glucose in the NHANES 1999–2012 dataset. A. Display of logistic regression illustrates that the probability of I-Hg detection in blood (above 0.4ug/L) is directly associated with fasting glucose levels in the raw NHANES population (Odds Ratio 1.02114, p=0.034, N=16,232). B. Same as A except this is a multivariate analysis that adjusts for race and sex (Odds Ratio 1.02886, p=0.004, N=16,232). C. Same as A except this is the survey weighted population (Odds Ratio 1.03395, p=0.006, N=16,232, SP=4.137e+08). D. Same as B except this is a survey weighted population (Odds Ratio 1.04260, p=0.001, N=16,232, SP=4.137e+08).

In the NHANES 1999–2012 dataset, n=16,232, we found a significant, direct relationship between blood inorganic mercury (IHg) and fasting blood glucose (Table 3). This was true of both the continuous variable for blood inorganic mercury (I-Hg concentration, p<0.001) and the binary variable, I-Hg detect (OR 1.03, p=0.006). After adjustment for age, race, and gender, I-Hg concentration remains significantly associated (p<0.001) with blood glucose levels in the survey-weighted population. Figure 1 shows these associations that were significant in the raw and survey weighted populations and after adjusting for age, race, and gender. Although blood organic mercury was associated with glucose levels (p<0.001), this correlation fell out and became not significant when adjusted for age, race and gender (p=0.097). However, blood organic mercury was inversely associated with insulin levels even after adjusting for age, race, and gender (p<0.001). Our results indicate that blood glucose and inorganic mercury (I-Hg) share an association that is unique to this mercury speciation. The inverse association between blood organic mercury with insulin suggests a complex relationship between the levels of different mercury forms, glucose and insulin.

### Results of Health Education Interventions

All ten participants received instruction on the macroepigenetic model for insulin resistance by attending the pre-intervention workshop or participating in the online course. Of the ten participants in the study, five who were not assigned to the CSE group successfully completed the online intervention course (MAC). Four of the five participants in the CSE group successfully eliminated corn sweeteners from their diet. There was one drop out in the CSE group. Blood samples were collected from all ten participants pre and post intervention(s) and analyzed for mercury as well as glucose and insulin to determine insulin resistance using the homeostasis model of assessment for insulin resistance (HOMA-IR). The data from the CSE group drop out was omitted and not included in the analysis of the results. In post-intervention interviews, this participant admitted she was unable to comply with the protocol and abstain from corn sweetener consumption.

### MAC intervention course

Pre-test blood samples were collected for analysis from the five intervention course participants the third week of January 2013 and post-test blood samples were collected for analysis the first week of April 2013. Matched data on dietary intake were also collected from the intervention course participants who completed the IRB approved food frequency survey questionnaire online at two points in time during the online macroepigenetics nutrition intervention course. The first pre-test survey was administered during the third week of January 2013. The second post-test survey was completed by the participants in the same manner at the end of the 10-week course.

The diet behavior score data derived from the embedded food frequency survey administered during the online macroepigenetics nutrition intervention course are provided in Table 2. An item was scored positively if the subject reported a diet habit consistent with the instruction. Higher scores indicate a healthier diet. The data show a significant difference between the pre and post periods in the category of questions used to measure participant consumption of whole foods with minimal processing and/or organic food products. The total pre- intervention diet score is 27 and the post- intervention diet score is 42. The t-test analysis was significant with p<0.01. These findings indicate the participants significantly increased their consumption of whole foods with minimal processing and/or organic food products. There was no change in the participants’ fish consumption, however, between the pre and post periods. In the category of questions used to measure fish consumption, the total pre-intervention diet score is 5 and the post intervention score is 6. The t-test analysis was not significant with p>0.05.

The diet score data in Table 2 also show a significant difference between the pre and post periods in the category of questions used to measure participant consumption of highly processed foods, excluding corn sweeteners and sugars. For the highly processed food category, the total pre-intervention diet score was 27 and the post-intervention diet score was 38. The t-test analysis was significant with p<0.01. The increase in this diet score indicates participants significantly reduced their consumption of highly processed foods. Participants also reduced their intake of foods comprised of corn sweeteners or high in sugar but there was not a significant difference in the pre and post-intervention diet scores for this category of questions. The total pre-intervention diet score is 13 and the post intervention score is 18. The t-test analysis was not significant with p>0.05. The increased score in this category, however, indicates the participants reduced their overall corn sweetener and sugar intake.

Table 4 shows a significant difference between the average fasting blood glucose measurements in the two periods. The average fasting blood glucose for the 5 matched subjects is 101.4 mg/dL in the pre- test period and 88 mg/dLin the post- test period at the end of the 10 week intervention period. The t-test analysis was significant with p<0.01. The analysis of the plasma insulin data show there is no significant difference between the pre and post test although there is a downward trend in the average insulin between the two time periods. The average fasting insulin for the 5 matched participants is 15.9 uU/ml in the pre test and 13.82 uU/ml in the post-test. The analysis of the HOMA-IR data show there is no significant difference between the pre and post test although there is a downward trend in the average HOMA-IR between the two periods. The average HOMA-IR for the 5 matched participants is 4.14 in the pre test and 2.98 in the post-test.

Table 4 also shows the analyses of the BMI and weight data. For both indices there is no significant difference between the averages in the two periods. Average BMI for the 5 matched participants is 29.44 in the pre test, and 28.8 in the post-test. The t-test analysis was not significant. The weight data show no significant difference between the pre and post test although there is a downward trend in the average weight between the two time periods. The average weight for the 5 matched participants is 194.24 pounds in the pre-test and 189.88 pounds in the post-test.

### CSE support group intervention

Matched data pre and post for the blood sample analyses were collected from all four participants in the CSE group who gave blood samples at two points in time during the 10-week support group intervention for the elimination of corn sweeteners. The first pre-test samples were collected before the first support group meeting during the third week of January 2013. The second post-test samples were collected the first week of April 2013 at the end of the intervention period.

Table 5 shows that there is significant difference between the average weight and BMI measurements in the two periods. The average weight for the 4 matched participants is 216.3 pounds in the pre-test period and 209.3 pounds in the post-test period. The t-test analysis was significant with p<0.01. The average BMI for the 4 matched participants is 33.45 in the pre-test period and 32.35 in the post- test period. The t-test analysis was significant with p<0.01. The participants who eliminated corn sweeteners from their diet showed significant losses in both weight and BMI.

Table 5 also provides the fasting glucose data that show no significant difference between the pre and post test although there is a downward trend in the average fasting glucose between the two time periods. The average fasting blood glucose for the 4 matched subjects is 97.75 mg/dL in the pre test period and 82 mg/dL in the post test period at the end of the 10 week intervention period. The t-test analysis was not significant. The analysis of the plasma insulin data show there is no significant difference between the pre and post test. The average fasting insulin for the 4 matched participants is 11.98 uU/ml in the pre test and 12.58 uU/ml in the post-test. The analysis of the HOMA-IR data show there is no significant difference between the pre and post test although there is a slight downward trend in the average HOMA-IR between the two periods. The average HOMA-IR for the 4 matched participants is 2.92 in the pre test and 2.76 in the post-test.

### Post intervention mercury (Hg) results

Post intervention Hg levels of the participants in the two intervention groups were analyzed and compared and the results are provided in Table 6. The blood samples were analyzed for total Hg, inorganic mercury (I-Hg) and methyl mercury. The mercury species found in the blood samples were exclusively inorganic mercury (IHg), and no methlymercury was detected in any of the blood samples analyzed at the sub-ng/g detection limit levels. The average total Hg level for the CSE group is 1.325 ng/g and the average total Hg level for the online MAC intervention course group is 0.332 ng/g. Although there is a large difference in the average Hg levels between the two groups, the difference is not significant with p=0.052. The difference in the post Hg levels between the two intervention groups and the reductions in fasting glucose levels observed in both intervention groups suggested inorganic Hg may play a role in glucose homeostasis.

## Discussion

Dietary behavior plays a significant role in determining risk of T2D and other chronic diseases in the AI population. Consumption of specific food ingredients can lead to obesity and increased risk of T2D. Goran, Ulijaszek and Ventura [55] found that diabetes prevalence was 20% higher in countries with higher availability of high fructose corn syrup (HFCS). Corn sweeteners, including HFCS have been pervasive as an ingredient available in the AI food supply since the 1970’s and may be a source of mercury exposure [31,32]. The Pima, an AI tribe in southern Arizona, have the highest prevalence of T2D in the world, yet their genetically related indigenous counterpart, living across the American border in Mexico; have at least 5 times less diabetes [56]. Esparza-Romero *et al.* [56] found that Mexican Pima with normal glucose levels have lower mean insulin resistance (HOMA-IR 1.40) compared to their AI counterpart (HOMA-IR 3.07), even after controlling for differences in obesity, age, and sex. The difference in HOMA-IR between groups could only be partially explained by the greater degree of obesity in the AI Pima [56]. One explanation for the greater obesity in the AI Pima could be their exposure to HFCS. HFCS was not available for consumption in Mexico during and many years prior to the study period [57]. Up until 2008, Mexico severely limited imports of HFCS to protect their sugar market [57].

HFCS consumption is thought to play a role in the obesity epidemic but the genetic mechanisms remain unclear [58]. Stanhope *et al.* [59] compared metabolic outcomes for two different groups of human subjects, one fed fructose sweetened beverages (n=17) and the other fed glucose sweetened beverages (n=15) for eight weeks. The fructose fed group significantly increased their belly fat compared to the glucose fed group and exhibited greater insulin resistance [59]. Fasting glucose levels also increased in the subjects fed fructose [59].

In the present study, the participants in the CSE group significantly reduced their weight and BMI with the elimination of fructose and other corn sweeteners from their diets. All had normal fasting glucose levels at the beginning of the study that decreased over the 10-week intervention period. A downward trend in the HOMA-IR value was also observed suggesting that with the loss in weight and BMI, the participants significantly reduced their risk of T2D. The elimination of corn sweeteners, including HFCS from the diet appears to contribute to the weight and BMI losses. With respect to mercury levels, the participants in the CSE group that eliminated corn sweeteners from their diet, had lower inorganic blood mercury than the one drop out in the CSE group who was unable to comply with the protocol. This individual had the highest post intervention mercury level of all at 5.01 ng/g.

In follow up interviews, the participants in the CSE group stated the task of eliminating HFCS and other corn sweetener from their diet was difficult. They felt HFCS was “addictive.” The addictive property of fructose has been compared to that of ethanol [60]. Although the restrictive diet was stressful, the participants in the CSE group felt their role in the study was important and wanted to succeed in their goal of eliminating HFCS and corn sweeteners from their diet. The participants stated they found the support group “helpful.” The weekly sessions enabled them to monitor their dietary intake to achieve their goal of eliminating HFCS and other corn sweeteners from their diets. Previous research shows that self-monitoring and goal-setting enhances self-efficacy and fosters behavior change beyond what is seen with just knowledge alone [61]. The results of this pilot study demonstrate the added value of goal setting and self-monitoring in a support group setting to knowledge gained through macroepigenetics workshop attendance.

The MAC intervention course was a successful tool in changing the dietary behavior of the participants in the study who took the online course. The curriculum provided a pathway of learning to improve student knowledge of toxic invasive substances in the food supply. Participants engaged in healthier eating habits as a result of the instruction. They significantly reduced their consumption of highly processed foods, invasive toxic substances, including I-Hg, which may be associated with the development of insulin resistance and T2D. The fasting glucose levels of the participants in the MAC intervention course significantly decreased along with their dietary changes that resulted in lower blood I-Hg levels compared to the CSE group.

This study is the first to show a direct relationship between blood inorganic Hg (I-Hg) and fasting blood glucose levels. The NHANES dataset analyses revealed this relationship is unique to blood I-Hg as glucose was not associated with blood organic mercury after adjustment for age, race, and gender. Blood organic mercury (CH_3_Hg) was inversely associated with insulin. It has previously been argued that blood I-Hg is the best biomarker found in NHANES for the assessment of I-Hg deposition in the body and toxic effect [62]. In contrast, blood organic (CH_3_Hg) and urinary mercury are measures of more recent exposure. Our results indicate recent organic mercury exposure affects insulin levels but not glucose levels, whereas chronic I-Hg exposure affects blood glucose levels. Following this logic, it may be inferred that I-Hg deposition due to chronic dietary mercury exposure has a direct relationship with blood glucose levels and may be a significant factor in the development of T2D.

I-Hg mercury may pass through the intestinal barrier into the blood stream via multiple mechanisms [63] depending on intestinal permeability. Teixeira *et al.* [64] describe the mechanisms that influence intestinal permeability in obesity to include: gut bacteria and other microbiota, high fructose and high fat diet, and nutritional deficiencies. Ingested food may be rich in sulfur or sulfhydryl-containing molecules [63]. These molecules bind easily to I-Hg [63] and are attached to proteins that may cross the intestinal barrier when it has become permeable by a high fat or high fructose diet. One sulfhydryl-containing protein that may play a role in the transfer of I-Hg across the intestinal barrier is serum albumin [63] which is endogenous to the intestinal tract [65]. In a recent study of the metabolism of ingested mercuric compounds, Yun *et al.* [66] determined the I-Hg mercury containing protein in human plasma is serum albumin. Once I-Hg is present in plasma, a component of blood, it may be taken up by the liver, kidney, and other organs [63]. Intestinal permeability should decrease with restricted high fructose and high fat dietary intake resulting in lower blood I-Hg levels.

Another possible mechanism for the intestinal absorption of I-Hg is dietary sodium intake from the consumption of highly processed foods. Evidence has shown that sodium ion concentration may increase I-Hg absorption from the gut [67]. As decreasing intake of processed foods should decrease sodium concentration, this may partially explain the lower blood mercury observed in the MAC group compared to the CSE group in this intervention study. Sodium intake from processed food consumption not only impacts intestinal permeability but also may decrease PON1 gene activity [68]. Dornas *et al.* [68] reported finding decreased PON1 activity in fructose-fed insulin resistant rats on a high-salt diet.

**Figure 2:**
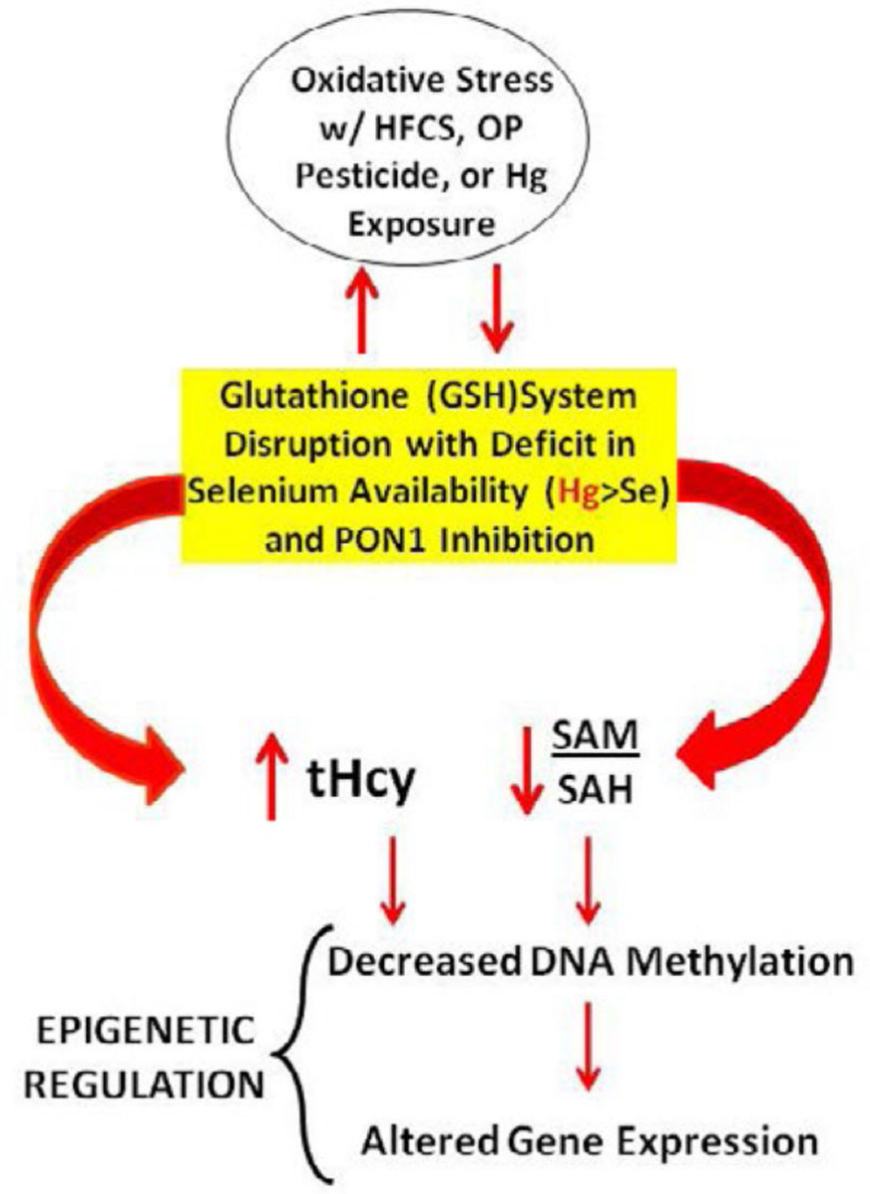
Dietary factors create oxidative stress and impact epigenetic regulation. **Legend**: Figure 2 shows the model that explains how PON1 gene status may be impacted by dietary factors high fructose corn syrup (HFCS) and inorganic mercury (Hg). When the diet is insufficient in selenium (Se) relative to Hg, there is disruption in the glutathione (GSH) system that creates oxidative stress. In the cycle of oxidative stress, a decrease in the S-adenosylmethionine (*SAM*) to S-adenosylhomocysteine (*SAH*) ratio results and this leads to decreased DNA methylation and altered gene expression. Inhibition of PON1activity undermines the body’s ability to metabolize organophosphate (OP) pesticide residues commonly found in grain and grain end products. This results in additional oxidative stress which may be marked by elevated total homocysteine levels.

PON1 gene status may play a role in obesity [69] independent of genetic ancestry [70] and lead to insulin resistance and diabetes [71]. Had this study included measures for PON1 gene expression, it likely would have found increased activity with the elimination of corn sweeteners, including HFCS, and I-Hg from the diet. PON1 gene activity can be modulated by fructose consumption [33, 22] and exposure to I-Hg [72]. I- Hg may interact with cysteine residues on PON1 preventing its activation in the liver and impairing the body’s ability to protect itself against oxidative stressors such as organophosphate (OP) pesticides [72]. PON1 is responsible for detoxifying homocysteine thiolactone [73] and essential for reducing total homocysteine (tHcy) levels which are thought to be harmful to health [17]. Total homocysteine (tHcy) levels are elevated in patients with T2D [74] and indicative if oxidative stress resulting from a reduction in PON1 activity. Ayotte *et al.* [7] found a significant inverse correlation between PON1 activity and total Hg levels and a direct correlation between PON1 activity and selenium (Se) levels in a study focused on the Inuit population. Sufficient dietary Se intake can offset the negative impacts of Hg exposure [7] with increased production of glutathione (GSH) peroxidase [75]. With deficits in dietary Se, the reverse is true. Chen *et al.* [76] found mercury exposure can affect the bioavailability and retention of selenium and interfere with the metabolic processes dependent on Se such as the GSH System. The model depicted in Figure 2 shows how epigenetic regulation may be impacted by these dietary factors and biochemical interactions. In the cycle of oxidative stress, a decrease in the S-adenosylmethionine (SAM) to S-adenosylhomocysteine (SAH) ratio results and this leads to decreased DNA methylation and altered gene expression [17].

**Figure 3:**
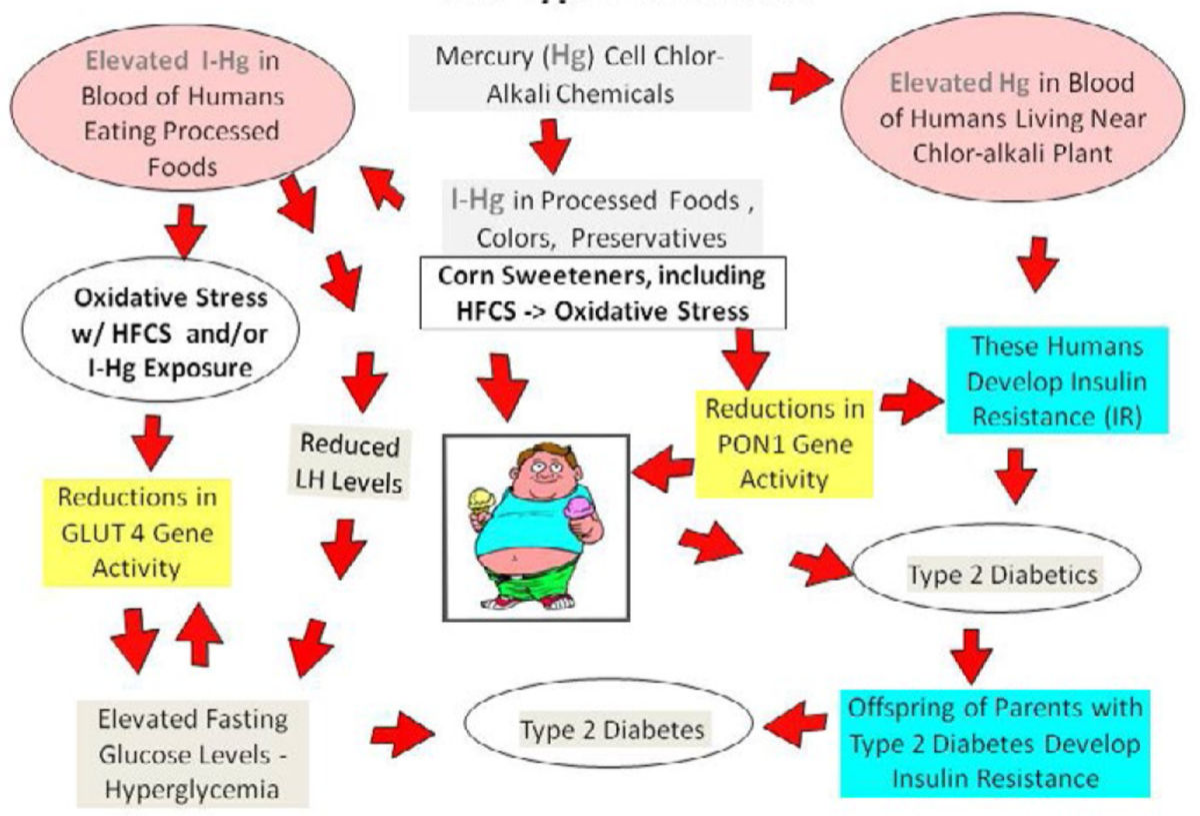
Macroepigenetic model of the role of I-Hg in glucose homeostasis and type-2 diabetes. **Legend**: The model is a flow chart of what can happen in the body when there is exposure to inorganic mercury (I-Hg) from the environment or ingestion of foods (via corn sweeteners such as high fructose corn syrup (HFCS), food colors, chlorinated flour, or other food ingredients processed with mercury (Hg) cell chlor-alkali chemicals). Glucose metabolism can be adversely impacted in three different ways including 1) when GLUT 4 gene expression is suppressed directly by inorganic mercury; 2) when GLUT 4 gene expression is suppressed indirectly by inorganic mercury from a reduction in luteinizing hormone (LH) levels and the resulting hyperglycemia; 3) via the oxidative stress created by inorganic mercury or HFCS exposure. Without proper GLUT 4 expression and regulation, glucose homeostasis may be disrupted resulting in elevated fasting glucose levels that could lead to the development of Type-2 Diabetes. Consumption of corn sweeteners may lead to reductions in PON1 gene expression and conditions for the development of obesity or insulin resistance leading to the eventual development of Type-2 Diabetes. Humans exposed to mercury may develop insulin resistance and Type-2 Diabetes. Offspring of parents with Type-2 Diabetics may suffer a transgenerational effect when they develop insulin resistance.

DNA methylation also plays a key role in the regulation of genes involved in glucose homeostasis [77]. It is not known, however, if DNA methylation is implicated in the down regulation of the GLUT4 gene [77]. GLUT4 plays a major role in regulating whole body glucose homeostasis [78, 79] and its expression has been found to be inversely associated with insulin resistance in obese diabetics [80]. I-Hg has been found to significantly reduce GLUT 4 gene activity altering glucose metabolism in adipocytes or fat cells [38]. Mercury exposure causes a modest increase in glucose transport and may contribute to the development of insulin resistance [39] by elevating glucose levels compared to insulin. In this study, the fasting glucose levels of the participants in the MAC intervention course significantly decreased along with their dietary changes that resulted in lower blood Hg levels compared to the CSE group. Had this study been able to monitor GLUT4 gene expression, it likely would have found an increase in GLUT4 activity with the dietary changes that were made by the participants in the MAC intervention group. The model presented in Figure 3 shows how GLUT4 gene expression can essentially be modulated by dietary conditions of inorganic mercury (I-Hg) exposure and/or reductions in the levels of luteinizing hormone (LH) [81].

The role of mercury in affecting LH levels has been studied by researchers. Previous reports have linked chronic I-Hg exposure with reduced levels of LH in both epidemiologic studies of the US population [45] and in toxicological studies of animals [82, 83]. LH is among the known regulators of insulin release in pancreatic cells [84]. In Figure 3, we show I-Hg exposure leading to reductions in LH creating conditions for the development of hyperglycemia which can further suppress GLUT4 gene activity [81]. Recent findings by Costanzo *et al.* [85] confirmed glucose levels negatively correlated with LH leading to hyperglycemia in men with T2D. As fasting glucose levels become elevated a negative feedback loop ensues contributing to further reductions in GLUT4 gene activity.

Figure 3 shows the model of the gene-environment interactions described in this discussion from a macroepigenetic point of view and illustrates the role of I-Hg exposure in glucose homeostasis, insulin resistance, and type-2 diabetes. It is important to note that Metformin is prescribed to patients with diabetes to control their glucose levels because of its ability to regulate GLUT4 activity [81,86]. Healthy dietary changes that reduce the intake of I-Hg, however, may have the same therapeutic affect on GLUT4 thereby reducing fasting glucose levels and the risk of T2D.

Mercury exposure has previously been identified as a factor in the development of T2D. He *et al.* [87] reported high mercury exposure in young adulthood likely contributed to elevated risk of diabetes later in life. The researchers followed 3,875 American healthy young adults, aged 20–32 years, for several years to determine what factors led to increased risk of diabetes. Two hundred eighty eight cases of diabetes were identified over 18 years of follow up [87]. The study [87] used multivariate analysis to adjust for age, sex, ethnicity, study center, education, smoking status, alcohol consumption, physical activity, family history of diabetes, dietary intake of long-chain n-3 fatty acids and Mg, and toenail Hg and Se. Baseline toenail Hg levels were measured using neutron-activation analysis and higher mercury exposures were associated with the development of diabetes later in life [87]. In a different study, researchers investigated 1,449 non-diabetic residents living near an abandoned mercury cell chlor-alkali plant and found residents with higher blood Hg *or* serum dioxins were at significantly higher risk for insulin resistance [40]. Abandoned mercury cell chlor-alkali plants release mercury into the air, soil, and water polluting the local environment for many years because treatment technologies are not yet available to clean up these facilities [88]. Mercury exposures in the residents living near the mercury cell chlor-alkali plant likely occurred through the consumption of contaminated fish caught locally, consumption of contaminated crops grown locally, inhalation of contaminated air, and ingestion of contaminated water [89]. Figure 3 shows these relationships.

Although fish and seafood consumption is thought to be the primary source of dietary mercury exposure, the results of the present study and recent evidence suggests this may not always be the case. From data derived from a food frequency questionnaire and blood samples collected from 4,484 pregnant women for mercury analysis, Golding *et al.* [90] estimated the contributions of 103 dietary variables to whole blood total mercury levels in the United Kingdom (UK). Although seafood was identified as a source of dietary mercury, it was determined to be a small proportion of the variation in the total blood mercury [90]. Maternal diet only accounted for 19.8% of the total variation in total blood mercury levels [90]. Forty four percent of the diet-associated variability was attributed to seafood consumption with the remaining diet-associated variability accounted for by wine and herbal teas, sunflower oil, bread, fresh fruit, and health food [90]. Golding *et al.* [90] did not consider HFCS as a source of mercury exposure in their study probably because it is not a significant food ingredient used in the UK due to trade restrictions. The results of the present study suggest that low level mercury exposure in the AI population living in the Fort Peck, Montana community occurs primarily through the consumption of highly processed foods including corn sweeteners.

The problem of heavy metal exposure from the consumption of food was emphasized recently in a study published by Kahn *et al.* [13]. Kahn *et al.* reported the concentrations of Cr, Cu, Mn, Ni, Pb, and Zn in food significantly correlated with the concentrations of the same metals found in the blood samples of the human subjects who consumed the food [13]. Higher dietary intake of heavy metals results in higher concentrations of heavy metals in blood and the associated changes in metabolism. For example, elevated serum concentrations of Pb and Hg were recently correlated with changes in diastolic blood pressure, fasting blood glucose, total cholesterol, triglycerides, and alanine aminotransaminase [91]. Other studies have also found associations between metals exposure and cardiometabolic risk [92], particularly with Hg exposure [93]. He *et al.* [87] reported higher organic Hg exposure at baseline was also significantly associated with decreased HOMA-B in the 3,875 American healthy young adults that were followed for many years to determine what factors lead to increased risk of diabetes. The results of the present study suggest I-Hg exposure may have a greater impact on HOMA-IR.

In the present study, average HOMA-IR values declined between the pre and post period in both intervention groups. HOMA-IR is calculated using a formula employing fasting insulin and glucose concentrations in plasma [94]. If insulin values remain constant, HOMA-IR increases in response to a rise in glucose levels. The findings of this study suggest that as I-Hg and fasting glucose levels increase so will HOMA-IR values. Chang *et al.* [40], in fact, found that HOMA-IR values increased for individuals with higher total blood mercury levels. The downward trend in the average HOMA-IR values in this study is a positive outcome for the AI participants, especially in the MAC intervention group with the pre test HOMA-IR value of 4.14 declining to the post test HOMA-IR value of 2.98. Zhang *et al.* [95] reported the risk of a cardiovascular disease event increased significantly in a nondiabetic AI population when HOMA-IR values ≥3.57. Interventions aimed at reducing blood mercury levels in the AI population should therefore reduce risk of cardiovascular disease.

This study did not attempt to determine the mercury exposure in the participants associated with dental amalgam. Elemental mercury exposure from dental amalgam is best determined by measuring urinary mercury levels [96]. Urine samples were not collected for mercury analysis as part of this study design. Fortunately, none of the participants in the study reported having undergone any dental procedures during the 10-week intervention period.

There is evidence to suggest the metabolic condition of insulin resistance may be passed on from diabetic parents to their offspring [97,98]. Figure 3 shows this transgenerational effect. In analyzing biomarker data collected by the US National Health and Nutrition Examination Survey (NHANES) from 6,174 women of childbearing age, Laks [45] found an upward trend in the blood inorganic Hg (IHg) levels. Within the population under study, detection of I-Hg levels rose sharply from 2% of the women in 1999–2000 to 30% in 20052006. It is therefore essential that a precautionary approach be taken and appropriate health education interventions focusing on dietary change be implemented to combat the obesity and T2D epidemics in the AI population. This study and successful alternative intervention approach is an example of the *precautionary principle* in action. The precautionary principle states “When an activity raises threats of harm to human health or the environment, precautionary measures should be taken even if some cause and effect relationships are not fully established scientifically [99].” Clearly the higher HFCS and processed food availability combined with preliminary studies raise a threat of harm to high HFCS and processed food consumers and intervention is appropriate. Recent understanding of the epigenetic changes induced by invasive toxic substances in the food supply indicates that dietary changes are appropriate.

Future intervention studies should include a significantly larger sample size. The findings of this small Phase I pilot study show there is a large effect and the intervention is ready for Phase II with more subjects and a control group. The future Phase II study should focus on changes in gene activity associated with the elimination of corn sweeteners, including HFCS, and highly processed foods from the diet. A better understanding of gene-environment interactions related to glucose homeostasis and the development of insulin resistance would likely be achieved and lead to continued improvements in health education programs designed to reduce risk factors for T2D.

## Conclusions

Community based participatory research aimed at testing dietary interventions to improve health outcomes can lead to significant findings even with small sample sizes when the findings can be validated by accessing public databases and analyzing variables of interest in larger datasets such as those found in NHANES. In this study, reductions in the consumption of processed foods led to the finding of a direct relationship between inorganic blood mercury and glucose in the human population. The design and content of the macroepigenetics nutrition intervention course played a role in helping the participants make diet behavior changes leading to significant reductions in fasting glucose and lower blood mercury levels compared to the participants who eliminated corn sweeteners, including HFCS from their diet. The CSE support group played a role in helping the participants make diet behavior changes leading to significant reductions in body weight and BMI. These changes in diet behavior, fasting glucose, weight and BMI likely reduced the participants’ risk of developing T2D.

The need to educate nutrition and health education professionals in this area is essential. Since dietitians, nutritionists, and health educators are the source of nutrition information for the public, they must have an understanding of this emerging field of macroepigenetics and, in particular, the macroepigenetic model for the role of I-Hg exposure in glucose homeostasis and T2D illustrated in Figure 3. Our findings suggest that chronic inorganic mercury exposure may adversely impact glucose homeostasis via different mechanisms thereby increasing the risk of T2D.

## Figures and Tables

**Table 1.** Change in American sugar and vegetable oil consumption 1970–2010.

Commodity	1970 per capita consumption (Lbs/year)	2010 per capita consumption (Lbs/year)	Percent increase or decrease
Cane and beet sugar	59.8	38.7	− 35%
High fructose corn syrup	0.3	28.7	+9,467%
Total corn sweeteners, including high fructose corn syrup	9.3	37.8	+306%
Vegetable oils (salad and cooking)	15.4	53.6	+ 248%

**Table 2.** Food frequency survey diet scores for MAC participants.

Category	Pre- test	Post- test	t-test
Questions	n=5	n=5	
**Corn sweeteners, refined or high sugar**			
How often do you drink a sugar sweetened beverage (do not include diet drinks)? ^a^	2	3	**p>0.05**
How often do you drink an energy drink? ^a^	4	4	
During the past month, how many times did you eat canned fruit (applesauce, apricot halves, mixed fruit, pears, cling peaches)? ^a^	5	5	
During the past month, how many times did you drink 100% fruit juice (apple, orange, grape, cranberry, other)? ^c^	1	1	
During the past month, how many times did you eat “sweet snacks” such as candy, cookies, ice cream, popsicle, other sugar sweetened treat (do not include diet)? ^d^	1	5	
Total score for category	13	18	
Mean	2.6	3.6	
SD	1.817	1.673	
**Fish**			**p>0.05**
How many times in the past month have you eaten freshly caught fish? ^b^	2	1	
During the past month, how many times did you eat canned tuna? ^b^	3	5	
During the past month, how many times did you eat canned salmon? ^c^	0	0	
Total score for category	5	6	
Mean	1.66	2	
SD	1.528	2.646	
**Whole foods (minimal processing) and/or organic products**			
During the past month, how many times did you eat fresh or frozen fruit (bananas, oranges, apples, strawberries, etc….)? ^e^	3	4	
During the past month, how many times did you eat fresh vegetables (spinach, lettuce, tomato, carrot, green salad, etc…)? ^e^	2	5	
**Whole foods (minimal processing) and/or organic products**			**p<0.01**
During the past month, how many times did you eat frozen vegetables (corn, broccoli, peas, green beans, etc….)? ^e^	2	3	
During the past month, how many times did you eat poultry (chicken or turkey)? ^e^	3	4	
How often in the past month, did you eat red meat (hamburger, pork, ham, or sausage)? ^d^ (moderation)	2	2	
During the past month, how many times did you eat “brown rice”? ^f^	3	4	
During the past month, how many times did you eat oats (oat meal)? ^f^	5	4	
During the past month, how many times did you eat canned vegetables (green beans, carrots, corn, peas, spinach, sweet potatoes, diced tomato, mixed vegetables)? ^b^ (moderation)	1	2	
How many times in the past month did you eat foods prepared with organic flour? ^f^	0	3	
How many times in the last month did you eat organic vegetables or fruit (fresh or frozen)? ^f^	4	4	
How many times in the past month did you eat organic processed foods (crackers, bread, cereal, canned vegetables, salad dressing, etc….)? ^f^	2	5	
Total score for category	27	42	
Mean	2.454	3.818	
SD	1.368	0.874	
**Highly processed foods**			**p<0.01**
During the past month, how many times did you eat canned meals (soup, re-fried beans, chili with and without beans, beef stew, etc..)? ^d^	5	5	
During the past month, how many times did you eat processed cheese (American)? ^a^	1	3	
During the past month, how many times did you eat processed meat (lunch meat, hotdogs, bacon, beef jerky, etc…)? ^d^	3	4	
During the past month, how many times did you eat ready-to-eat cereal (corn flakes, rice crisp, corn squares, oat circles, etc….)? ^d^	5	5	
During the past month, how many times did you eat foods fried in vegetable oil, lard, or butter, such as potato chips, french fries, fry bread, doughnuts, hash browns, fried eggs, etc…? ^d^	1	3	
During the past month, how many times did you eat “salty” snacks such as potato chips, pretzels, corn chips, pop-corn, etc…? ^d^	3	5	
During the past month, how many times did you eat grain products made of wheat such as macaroni, bread, hamburger buns, hotdog buns, or spaghetti? ^d^	2	3	
During the past month, how many times did you eat “white” rice? ^d^	4	5	
During the past month, how many times did you eat meals prepared in restaurants, fast food places, pizza parlors, or from vending machines? ^d^	3	4	
Total score for category	27	38	
Mean	3	4.222	
SD	1.414	0.972	

a score = 1 if “never, rarely (once or twice a month)”

b score = 1 if “once a week, rarely (once or twice a month)”

c score = 1 if “once a week, several times a week, every day (1–2 servings)”

d score = 1 if “never, rarely, once a week”

e score =1 if “several times a week, pretty much every day (1–2 servings), several times a day (3 or more servings)”

f score = 1 if “rarely (once or twice a month), once a week, several times a week, pretty much every day (1–2 servings)”

**Table 3.** Mercury (Hg) as a function of glucose (mM) in NHANES 1999–2012 Survey Weighted Population.

Population	Hg Species	Outcome	Regression	Correlation	Confidence	P-value
N=16,232, SP=4.137e+08	Blood I-Hg^a^	Glucose (mM)	Linear	β =0.00260	CI (0.00234–0.00286)	p<0.001
N=16,232, SP= 4.137e+08 Adj. for age, gender, race	Blood I-Hg^a^	Glucose (mM)	Linear	β =0.00150	CI (0.00123–0.00178)	p<0.001
		MA (vs CAU)	Linear	β =0.00184	CI (0.00078–0.00289)	p=0.001
		Black (vs CAU)	Linear	β =0.00394	CI (0.00312–0.00475)	p<0.001
N=16,232, SP=4.137e+08	Blood I-Hg^b^	Glucose (mM)	Logistic	OR=1.03395	CI (1.00980–1.05867)	p=0.006
N=16,232, SP=4.137e+08 Adj. for race and gender	Blood I-Hg^b^	Glucose (mM)	Logistic	OR=1.04260	CI (1.01674–1.06911)	p=0.001
		MA (vs CAU)	Logistic	OR=0.70323	CI (0.58613–0.84372)	p<0.001
N=16,230, SP=4.137e+08	Blood Organic Hg^a^	Glucose (mM)	Linear	β = 0.05923	CI (0.03654– 0.08193)	p<0.001
N=16,230, SP=4.137e+08 Adj. for age, gender, race	Blood Organic Hg^a^	Glucose (mM)	Linear	β = 0.01952	CI (−0.00364– 0.04268)	p=0.097
N=16,230, SP=4.137e+08	Blood Organic Hg^a^	Insulin (pM)	Linear	β = - 0.00121	CI (−0.00165– −0.00076)	p<0.001
N=16,230, SP=4.137e+08 Adj. for age, gender, race	Blood Organic Hg^a^	Insulin (pM)	Linear	β = −0.00120	CI (−0.00161– −0.00078)	p<0.001

N = Raw Population, SP = Survey Population, Adj = adjusted, I-Hg = Inorganic Mercury, ug/L = micrograms/liter, mM = millimolar, β = coefficient,

a refers to concentration of mercury(ug/L),

b refers to detection above 0.4 ug/L blood I-Hg, CI = Confidence Interval, MA = Mexican American, CAU = Caucasian, OR = Odds Ratio, pM = picomolar

**Table 4.** Changes in health status observed in macroepigenetics nutrition intervention course (MAC) group.

Online macroepigenetics nutrition intervention course (MAC) Group	Pre n=5	Post n=5	p-value
Mean weight (in pounds) SD	194.24 59.90	189.88 55.31	
Mean BMI SD	29.44 5.76	28.8 5.17	
Mean fasting glucose (mg/dL) SD	101.4 8.14	88 8.40	**<0.01**
Mean insulin (uU/ml) SD	15.9 13.32	13.82 11.46	
Mean HOMA-IR SD	4.14 3.64	2.98^a^ 2.40	

BMI = Body Mass Index, SD = Standard Deviation, HOMA-IR = Homeostasis Model of Assessment for Insulin Resistance,

a Lower HOMA-IR values significantly reduce risk for cardiovascular disease event [95].

**Table 5.** Changes in health status observed in corn sweetener elimination (CSE) group.

Corn sweetener elimination (CSE) group	Pre n=4	Post n=4	p-value
Mean weight (in pounds) SD	216.3 60.03	209.3 60.22	**<0.01**
Mean BMI SD	33.45 8.27	32.35 8.29	**<0.01**
Mean fasting glucose (mg/dL) SD	97.75 11.62	82 11.40	
Mean insulin (uU/ml) SD	11.98 4.89	12.58 7.31	
Mean HOMA-IR SD	2.92 1.34	2.76 1.77	

BMI = Body Mass Index, SD = Standard Deviation, HOMA-IR = Homeostasis Model of Assessment for Insulin Resistance

**Table 6.** Difference in mercury (Hg) levels post intervention.

Intervention group	Mean total post mercury (Hg) level (ng/g)	SD	p-value
Corn sweetener elimination group (CSE, n=4)	1.325	0.849	0.052
Online macroepigenetics nutrition intervention course (MAC, n=5)	0.332	0.100	

## List of abbreviations

NHANES: National Health and Nutrition Examination Survey
BMI: body mass index
AI: American Indian
AN: Alaska Native
USA: United States of America
T2D: type-2 diabetes
Cr: chromium
Cu: copper
Mn: manganese
Ni: nickel
Pb: lead
Zn: zinc
Mg: magnesium
P: phosphorus
HFCS: high fructose corn syrup
PON1: paraoxonase gene
FPCC: Fort Peck Community College
HgCl_2_: mercuric chloride
I-Hg: inorganic mercury
LOD: limit of detection
MAC: macroepigenetics nutrition intervention course
CSE: corn sweetener elimination support group intervention
IRB: institutional review board
FIHRI: Food Ingredient and Health Research Institute
NEMHS: Northeast Montana Health Services
USDA: United States Department of Agriculture
HOMA-IR: homeostasis model of assessment for insulin resistance
Hg: mercury
CDC: Centers for Disease Control
EPA: Environmental Protection Agency
D-IDMS: direct isotope dilution mass spectrometry
ICP-MS: inductively coupled plasma mass spectrometry
CH_3_Hg: methylmercury
SIDMS: speciated isotope dilution mass spectrometry
GC: gas chromatograph
RCRA: Resource Conservation and Recovery Act
SD: standard deviation
OP: organophosphate pesticide
tHCY: total homocysteine
Se: selenium
GSH: glutathione
SAM: S-adenosylmethionine
SAH: S-adenosylhomocysteine
GLUT4: gene involved in glucose homeostasis
LH: luteinizing hormone
UK: United Kingdom
